# Effects of Embedded Helium on the Microstructure and Mechanical Properties of Erbium Films

**DOI:** 10.3390/nano9111564

**Published:** 2019-11-04

**Authors:** Wenbo Fu, Huahai Shen, Liqun Shi, Xiaosong Zhou, Xinggui Long

**Affiliations:** 1 Institute of Nuclear Physics and Chemistry, China Academy of Engineering Physics, Mianyang 621900, China; mike_fu@126.com (W.F.); zlxs77@126.com (X.Z.); 2 Applied Ion Beam Physics Laboratory, Institute of Modern Physics, Fudan University, Shanghai 200433, China; lqshi@fudan.edu.cn

**Keywords:** helium bubbles, sputtering, erbium film, mechanical properties, TEM

## Abstract

A series of helium (He) charged nanograin-sized erbium (Er) films were deposited by direct current (DC)-magnetron sputtering with different He/Ar mixture gases. The microstructure and mechanical properties of He-charged Er films were investigated by X-ray diffraction (XRD), transmission electron microscopy (TEM), and nanoindentation. The helium concentrations in Er films, determined by elastic recoil detection analysis (ERDA), ranged from 0 to 49.6%, with the increase in He:Ar flow ratio up to 18:1. The XRD results show that the grain sizes of Er films decreased with and increase in He content. The embedded He atoms induced the formation of spherical nanometer He bubbles, and the diameter of the He bubbles increased with the He content. The hardness and Young’s modulus increased and decreased with the decreasing grain sizes of polycrystalline Er–He films. The mechanisms of mechanical properties with respect to the grain size and He content were discussed based on the Hall–Petch formula and composite spheres model.

## 1. Introduction

Erbium is a vital candidate material for tritium storage in solid phase [1], due to its higher thermal stability of its tritide and better accommodation ability for helium atoms generated by the decay of tritium [2]. The He bubble evolution-induced mechanical property degradation impedes its application in the field of nuclear industry. The inert gas He atoms are insoluble in the metal matrix, which accumulate and form bubbles at vacancies, dislocations, or grain boundaries [3,4,5]. The evolution of bubbles will lead to variation in the microstructure and degradation of mechanical properties in the metal matrix [6,7]. The nucleation, growing, and interacting of helium bubbles in the metal matrix attract much attention from investigators [8,9]. Snow et al. [2] systematically studied the mechanism of ^3^He bubble growth by transmission electron microscopy (TEM) with the aging of Er tritide up to the ^3^He content of 0.37. It was found that the He bubbles grow as Griffith cracks [10] until the ^3^He content of ~0.15, while the bubbles grow in thickness by punching dislocation dipoles with further aging [2,6]. Shen et al. [11] and Bufford et al. [12] investigated the microstructure changes of Er and Er deuteride under He ion implantation and found that the He implantation induced the severe lattice expansion for (200) crystal plane.

The evolution of mechanical properties with the aging of Er tritide has been studied by Knapp et al. [8]. They demonstrated that the hardness evolution of Er tritides strongly related to He bubble growth behavior. A substantial increase in Er tritide hardness occurred when the ^3^He content was lower than 0.139, and showed a modest decrease during the subsequent aging [8]. The relationship between the mechanical properties and the He bubble evolution in Er and Er tritide with He content higher than 0.32 was not established due to the aging period being too long to prepare such samples. Generally, helium atoms can be embedded into metal matrixes and their tritides in different ways. One way is the accumulation of ^3^He through the radioactive decay of tritium in metal tritides [13,14]. The other way is the injection of He into materials by accelerator or ion implanter [11,15,16,17]. However, the aging period of Er tritide and the ion irradiation-induced artificial defects might affect the research efficiency and accuracy of He bubble evolution.

Shi et al. [18] and Wei et al. [19] proposed a new approach, that the helium atoms can be embedded into the metal films by direct current (DC)-magnetron sputtering under a mixture of He/Ar gases [20,21]. The Ti films with a He/Ti atomic ratio as high as 56 at.% were fabricated without artificial defects [18]. In this way, the amount of embedded helium could be well regulated and controlled, as well as the free of irradiation damage in the metal matrix in the implantation processing. In this study, helium atoms with different flow ratios of He/Ar were embedded into Er films by DC-magnetron sputtering under a mixture of He/Ar gases. The effects of the embedded He atoms on the microstructures and mechanical properties of Er–He films were studied by X-ray diffraction (XRD), TEM, elastic recoil detection analysis (ERDA), and nanoindentation.

## 2. Experimental Details

### 2.1. Sample Preparation

The Er films containing embedded He atoms were deposited onto polycrystalline molybdenum substrates in a DC-magnetron sputtering system. A pure Er disk (60 mm in diameter and 5 mm in thickness) with purity of a 99.9% was used as the sputtering target. The substrates were placed on a disc holder with a diameter of 70 mm, and a bias voltage of −300 V was applied to the holder. All experiments were performed at room temperature. The base vacuum before the deposition was better than 2 × 10^−4^ Pa, and the gas environment used during deposition was a mixture of high-purity argon (99.99%) and helium (99.99%). The working pressure was controlled to keep plasma stable and the relative flow ratio of helium to argon was varied to obtain Er films with different He contents. To increase the mean free path of sputtering atoms, a low total sputtering pressure with high He partial pressure was maintained. However, the argon partial pressure and the sputtering current were set to the minimum to sustain discharge. During the deposition, the argon partial pressure was from 0.9 to 1.5 Pa. The deposition time for all experiments was fixed at 39 min. The step profiler was used to analyze the thickness of Er films after deposition. The deposition rate was roughly calculated by dividing the film thickness by the deposition time.

### 2.2. Sample Characterizations

Elastic recoil detection analysis (ERDA) was performed to measure the depth distribution of helium atoms in the Er films. The ERDA measurements were explored at the NEC 9SDH-2 tandem accelerator [22] (Fudan University, Shanghai, China) in the Institute of Modern Physics of Fudan University using an 8 MeV^12^ C^3+^ ion beam source. The sample was tilted at 75° against the normal incidence in order to optimize the sensitivity and depth resolution. To avoid detecting scattered particles, a 10 µm thick mylar foil was positioned between the sample and the detector to absorb the scattered He. All the ERDA spectra were transformed into the depth distribution of helium concentration in Er films by Alegria 1.2 codes [23].

The crystal structure of Er films was examined by X-ray Diffraction (PANalytical B.V. Inc., Almelo, The Netherlands). The diffracto-meter worked in 2*θ* mode with 2*θ* varied from 20° to 90° by a 0.04° step, and used a Cu K α source. Mechanical properties were characterized by the nanoindentation using an Agilent G200 Nanoindenter (Agilent Inc., Santa Clara, CA, USA). The nanoindenter used a Berkovich diamond tip with a radius of about 50 nm. The helium bubble morphology and distribution were observed using a FEI Tecnai F20 TEM (Thermo Fisher Scientific Inc., Hillsboro, OR, USA) working at 200 kV. The TEM specimens were prepared by a focused ion beam method using a Zeiss Auriga workstation (Carl Zeiss Microscopy GmbH Inc., Jena, Germany).

## 3. Results and Discussions

### 3.1. Helium Depth Distribution in Er Films

Table 1 shows the mixture of sputtering gases (Q_He_:Q_Ar_ flow ratio) during deposition, the thickness of erbium films, and the He concentration in the Er films. As is well known, the Ar flow contributes more to the deposition rate of Er film, since the sputtering yield of Er atoms by Ar ions is much higher that by He ions. In order to maintain the proper sputtering pressure, the Ar partial pressure decreases with an increase in Q_He_:Q_Ar_ flow ratio. Therefore, the deposition rates of He embedded Er films can be adjusted by the Q_He_:Q_Ar_ flow ratio, and an increase in Q_He_:Q_Ar_ flow ratio leads to an obvious decrease in film thickness.

**Figure 1:**
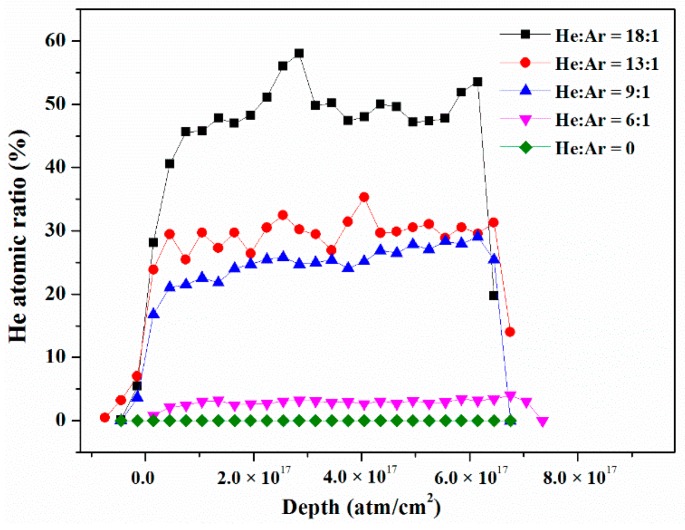
Helium depth distribution in Er films determined by ERDA.

Figure 1 shows the helium depth distribution of the Er films that was determined by the ERDA method. An enormous number of helium atoms were distributed thoroughly through the entire Er films and a maximum He concentration of 49.6 was found in the Er film with a Q_He_:Q_Ar_ flow ratio of 18:1. The atomicratio of embedded helium increased significantly with an increase in He/Ar flow ratio, which was ascribed to the following two major reasons. Firstly, the increasing helium flux induced the increase in helium ion concentration in the plasma. The helium atoms trapped and residing in the Er films came from the backscattered helium atoms from the target and helium ions in the plasma of the anode sheath layer. Therefore, the increasing helium ion concentration certainly would cause an increase in helium atom trapping in the growing Er film [24]. Secondly, helium atoms were adsorbed on the pure Er film surface by a binding energy of 0.2 eV, and had an average staying period of less than 10^−9^ s on the Er surface at room temperature. It is easy for He atoms to desorb from the near surface of thin films. Only those He atoms or ions with higher energy could be injected into the deeper area and were trapped in interstitial sites or defects in the crystal.

### 3.2. Microstructures of He-Embedded Er Films and the Morphology of He Bubbles in the Films

**Figure 2:**
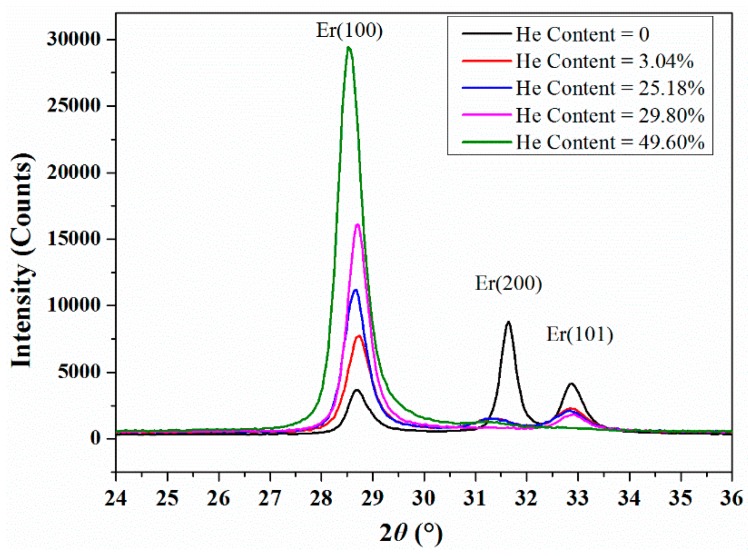
X-ray diffraction (XRD) patterns of the Er films deposited with different flow ratio of Q_He_/Q_Ar_.

Figure 2 displays the XRD patterns of the pure and He-charged Er films. In the pure Er film, the Er diffraction peaks of (100), (002), and (101) crystalline planes were positioned at 2*θ* values of 28.7°, 31.7°, 32.9°, 62.0°,and 66.0°. The pure Er film presented a preferred orientation of (002) plane. However, in the He-embedded Er films, the peaks with maximum intensity changes to the Er (100) and the other diffraction peaks decreased gradually with the increasing Q_He_:Q_Ar_ flow ratio. It was hard to recognize the Er diffraction peaks of (002) and (101) when the flow ratio of He:Er increased up to 29.8%. The Er preferred orientation of (100) was most obvious when the flow ratio of He:Er reached the maximal value of 49.6%.

Table 2 shows the 2*θ* values of Er diffraction peaks with different He concentrations. The Er (100) plane grew faster in the mixture gas environment of He and Ar than that in the pure Ar gas. The Er(100) peaks shifting to lower angle imply that the lattice expanded with the increasing flow ratio of He:Ar. The full width at half maxima (FWHM) of Er(100) peaks became broader with the increasing He concentration, indicating that the grain size of the Er films became smaller during the high flow ratio of He:Ar. The embedded He atoms distributed at the sites of defects and along grain boundaries in the Er films, which induced the increasing inner stress by stronger lattice expansion and inhibited the grain growth [19]. Consequently, the Er diffraction peak of (100) shifted and broadened with the increasing He concentration.

**Figure 3:**
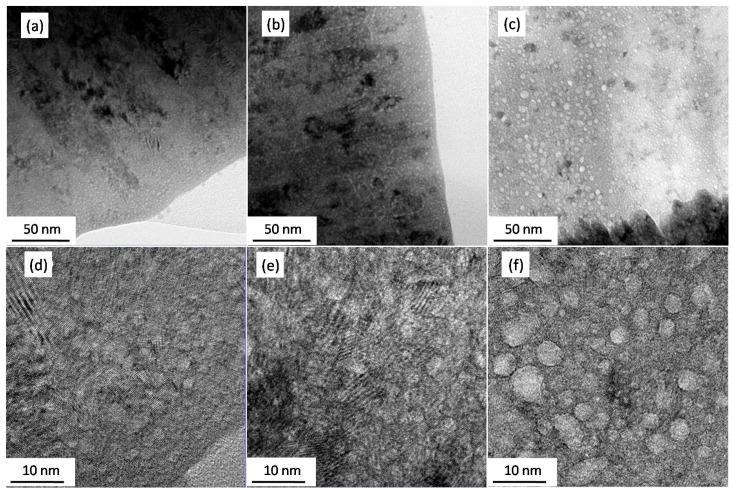
(BF) TEM images of Er film with He concentration of 3.04% (**a**,**d**), 29.81% (**b**,**e**), and 49.60% (**c**,**f**). The low magnification TEM images in (**a**–**c**) were enlarged to be shown in (**d**–**f**), respectively.

Figure 3 exhibits the typical bright-field (BF) TEM images of the as-deposited He embedded Er films with different He concentrations. A great many small white spots with diameters about 2–3 nm are visible in Figure 3a–c. The small white spots appear dark in the over-focus condition, but bright in the under-focus condition, which confirms the formation of He bubbles in the Er films. The sizes of He bubbles were analyzed by nano-measure software. In Figure 3d–f, the averagediameter of the spherical helium bubbles can be seen clearly. Interestingly, some of the bubbles presented as an elliptical shape, which might be due to the coalescence of the neighboring He bubbles or controlled by the Er crystal structure. The average sizes of bubbles rose from 2.1 ± 0.4 nm to 2.6 ± 0.4 nm and 3.8 ± 1.1 nm with the He concentration increase from 3.04% to 29.81% and 49.6%.

### 3.3. Mechanical Propertiesof He-Embedded Er Films

As shown in the inset of Figure 3b, a series of polycrystalline rings were observed in the selected area electron diffraction pattern, which implies the nanocrystalline feature of Er film with embedded He atoms. It could also be calculated from the XRD diffraction peak that the grain size of He-embedded Er films decreased with the increase in He concentration from 19.7 nm to 14.9 nm. It is well known that the hardness of nano-sized thin film would be strengthened by grain refinement, based on the traditional Hall–Petch mechanism [25,26]. The grain size is considered to be the major factor affecting the hardness of polycrystalline material. The relationship between the hardness and grain size could be expressed by the classical Hall–Petch formula:

*σ_y_* = *σ*_0_ + *Kd*^−1/2^, (1)

where *σ_y_* is Brinell hardness, *σ*_0_ and *K* are both constant, *d* is the grain size [26,27]. For pure Er film, the *σ*_0_ was calculated to be 0.512 GPa.

**Figure 4:**
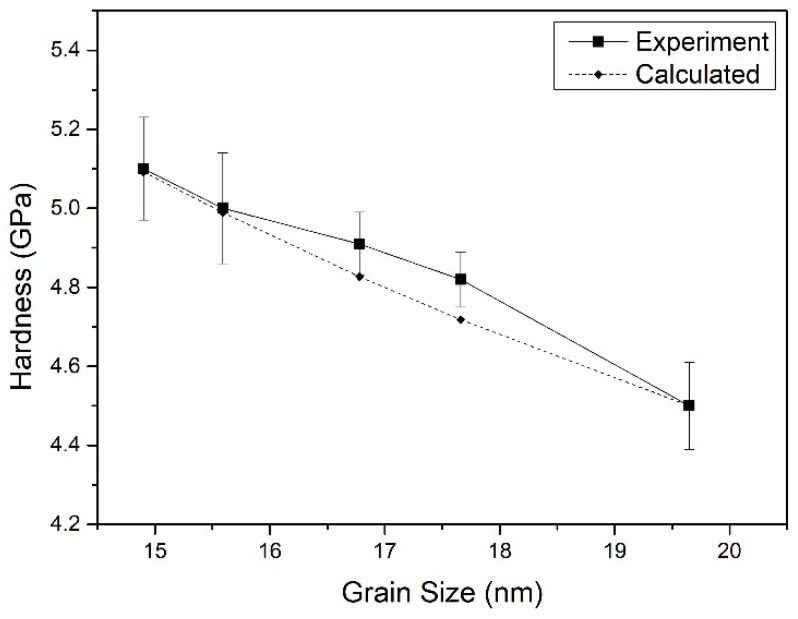
Tendency of the hardness of Er films with grain sizes.

Figure 4 shows the experimental values of the hardness of Er films measured by nanoindenter and the corresponding theoretical values calculated by the Hall–Petch formula. It is clear that the hardness of He-embedded Er films decreased with the increase in Er grain size that conformed to the relationship between the hardness and grain size predicted by Equation (1). The pure Er film with the largest grain size had the lowest hardness of 4.5 GPa. The hardness of Er films with lower He concentrations of 3.04% and 25.18% increased to 4.91 GPa and 4.82 GPa when their grain sizes reduced to 17.7 nm and 16.8 nm, respectively. Those are a little higher than the calculated values. When the grain sizes of He-embedded Er films decreased down to 15.6 nm and 14.9 nm, the hardness values almost increased up to 5.00 GPa and 5.10 GPa. The changing tendency of Er film hardness with the Er grain size was well fitted by the Equation (1), except for the little higher experimental values than those calculated ones at the grain sizes of 17.7 nm and 16.8 nm. It can be concluded that the grain size plays an important role in the hardness of nanocrystalline Er films with embedded He atoms. Additionally, the hardness of Er film is indirectly affected by the He atoms or bubbles, since the grain size of Er film has a strong relationship with the He concentration. The mechanical properties of the helium-embedded nanocrystalline metals are not only depended on the average grain size, but are also influenced by the inert gas vacancies [28,29]. In the low inert gas concentration films, the vacancies easily piled up at the grain boundaries and caused an enhancement of plastic resistance.

The Young’s modulus of the He-embedded Er films was characterized by the continuous stiffness method (CSM). The He volume fractions were calculated by multiplying the He bubble density by the average He bubble diameter. The elastic behavior of Er film with embedded He bubbles can be discussed based on the two phase composites model proposed by Christensen [27], which was derived for small spherical precipitates. Based on the composite spheres model [8,27], the effective Young’s modulus, *E*, could be calculated by the formula:

(2) $$E=E_{m}+\frac{f(E_{i}-E_{m})}{1+\frac{\left(1-f)(E_{i}-E_{m}\right)}{E_{m}+4G_{m}/3}},$$

where *E_i_* is the Young’s modulus of the spherical inclusion and *E_m_* is that of the film matrix. *G_m_* is the shear modulus of the film matrix, and *f* is the volume fraction of the spherical inclusion. The Young’s modulus of the film matrix was calculated by the formula:

(3) $$E_{m}=(1-\nu_{m})G_{m},$$

where ν_m_ is Poisson’s ratio of the matrix material:

(4) $$a=\frac{4}{3(1-v_{m})}.$$

As for the Er:He composite, *a* should be a constant since the ν_m_ is constant. Therefore, Equation (2) can be simplified as:

(5) $$E=aE_{m}\left(\frac{1-f}{a+f}\right).$$

**Figure 5:**
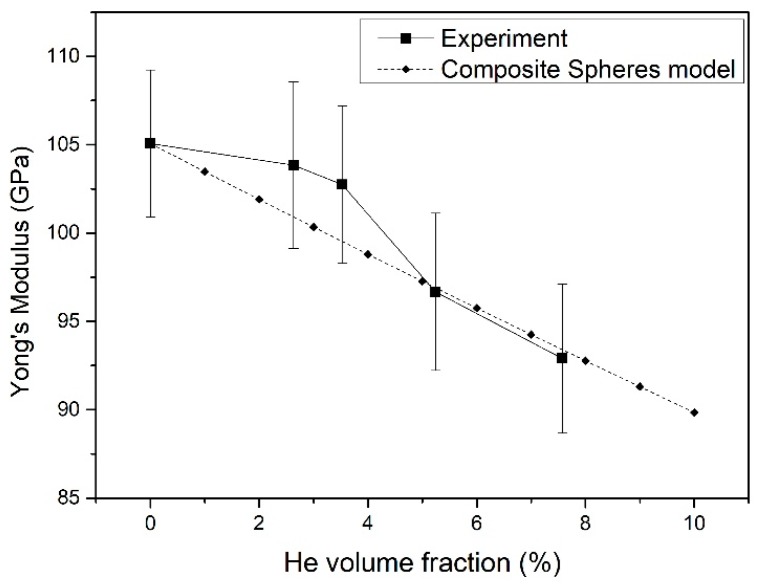
Relationship of Young’s modulus of Er films with respect to the He volume fraction.

In this work, the value of Poisson’s ratio used for Er was 0.3 and the calculated *a* was 1.9, based on Equation (4). The Young’s module of pure Er film was calculated as 105.07 Gpa. The He volume fraction could be estimated from the volume of a single He bubble and He bubble density in a selected area from the TEM images. The He volume fraction should be proportional to the He concentration in the Er films. Figure 5 depicts the curve of Young’s modulus versus the He volume fraction. Young’s modulus dropped gradually with the increase in He volume fraction. The experimental changing tendency of Young’s modulus with the He volume fraction was in accord with the calculated data based on the composite spheres model. It was similar to that of hardness, and the experiment values were a little higher than the calculated data for the lower He volume fraction of the 2.6% and 3.5% samples. It can be concluded that the Young’s modulus has a tendency to decrease with an increase in He content, which is in contrast to that of Er film hardness.

## 4. Conclusions

The He-embedded Er films were deposited onto Mo substrates by DC-magnetron sputtering under different He/Ar mixture gas environments. The ERDA results confirmed that the He concentrations in the Er films increased with the increasing flow ratio of He:Ar, and reached a maximum value of 49.6% at 18:1 of He:Ar. The aggregation of the embedded He atoms induced the formation of nano-sized He bubbles at a relatively high He concentration in the polycrystalline Er films. The increase in He:Ar flow ratio in the gas mixture led to grainrefinement, since they formed spherical He bubbles at the grain boundary, inhibiting the Er grain growth. The hardness increased with the decreasing grain sizes of polycrystalline Er–He films, which could be well explained based on the Hall–Petch mechanism that the hardening was correlated to grain refinement in the nanocrystalline thin film. However, Young’s modulus tended to decrease with the decreasing grain sizes of Er–He films, since the increasing fraction of He bubbles resulted in the elastic softening of He-embedded Er films. The major contribution of this study is proposing procedure for preparing Er films with an extra high He concentration of 49.6%, which is beneficial for shortening the research period of investigating the He bubble behavior in Er tritide after several years of aging.

## Figures and Tables

**Table 1:** The Er film deposition parameters and the measured He concentration in Er films by elastic recoil detection analysis (ERDA).

Flow Ratio of He:Ar	Deposited Rate /(nm/min)	Film Thickness /(µm)	He Concentration /(%)
0:1	60.0	1.80	0
6:1	48.0	1.44	3.04
9:1	42.7	1.28	25.18
13:1	42.3	1.27	29.80
18:1	31.7	0.95	49.60

**Table 2:** The (100) diffraction peak of Er films with different He concentrations.

He:Ar	He Concentration /(%)	2*θ* of (100) Peak /(°)	Normalized Peak Height/(%)	FWHM /(°)	Grain Size/(nm)
0:0	0	28.77	31.37	0.445	19.7
6:1	3.04	28.80	23.29	0.459	17.7
9:1	25.18	28.76	63.71	0.483	16.8
13:1	29.81	28.68	49.07	0.520	15.6
18:1	49.60	28.64	171.41	0.544	14.9
